# The paradoxical patterns of expression of indoleamine 2,3-dioxygenase in colon cancer

**DOI:** 10.1186/1479-5876-7-71

**Published:** 2009-08-20

**Authors:** Yan-Fang Gao, Rui-Qing Peng, Jiang Li, Ya Ding, Xing Zhang, Xiao-Jun Wu, Zhi-Zhong Pan, De-Sen Wan, Yi-Xin Zeng, Xiao-Shi Zhang

**Affiliations:** 1 State Key Laboratory of Oncology in South China, 651 Dongfeng R E, 510060, Guangzhou, PR China; 2 Biotherapy Center, Cancer Center, Sun Yat-sen University, 651 Dongfeng R E, 510060, Guangzhou, PR China; 3 Department of Medical Oncology, Weifang People's Hospital, 151 Guangwen Street, Kuiwen District, 261040, Weifang, PR China; 4 Department of Abdominal Oncology, Cancer Center, Sun Yat-sen University, 651 Dongfeng R E, 510060, Guangzhou, PR China

## Abstract

**Background:**

One of the putative mechanisms of tumor immune escape is based on the hypothesis that carcinomas actively create an immunosuppressed state via the expression of indoleamine 2,3-dioxygenase (IDO), both in the cancer cells and in the immune cells among the tumor-draining lymph nodes (TDLN). In an attempt to verify this hypothesis, the patterns of expression of IDO in the cancer cells and the immune cells among colon cancers were examined.

**Methods:**

Seventy-one cases of pathologically-confirmed colon cancer tissues matched with adjacent non-cancerous tissues, lymph node metastases, and TDLN without metastases were collected at the Sun Yat-sen Cancer Center between January 2000 and December 2000. The expression of IDO and Bin1, an IDO regulator, was determined with an immunohistochemical assay. The association between IDO or Bin1 expression and TNM stages and the 5-year survival rate in colon cancer patients was analyzed.

**Results:**

IDO and Bin1 were detected in the cytoplasm of cancer cells and normal epithelium. In primary colon cancer, the strong expression of IDO existed in 9/71 cases (12.7%), while the strong expression of Bin1 existed in 33/71 cases (46.5%). However, similar staining of IDO and Bin1 existed in the adjacent non-cancerous tissues. Among the 41 cases with primary colon tumor and lymph node metastases, decreased expression of IDO was documented in the lymph node metastases. Furthermore, among the TDLN without metastases, a higher density of IDO^+^cells was documented in 21/60 cases (35%). Both univariate and multivariate analyses revealed that the density of IDO^+^cells in TDLN was an independent prognostic factor. The patients with a higher density of IDO^+^cells in TDLN had a lower 5-year survival rate (37.5%) than the cells with a lower density (73.1%).

**Conclusion:**

This study demonstrated paradoxical patterns of expression of IDO in colon cancer. The high density IDO^+^cells existed in TDLN and IDO was down-regulated in lymph nodes with metastases, implying that IDO in tumor and immune cells functions differently.

## Background

Most dietary tryptophan enters the kynurenine pathway, leading to the biosynthesis of NAD or resulting in the complete oxidation of amino acids for energy production. Indoleamine 2,3-dioxygenase (IDO) and tryptophan 2,3-dioxygenase (TDO) can both function as the initial rate-limiting enzymes in the kynurenine pathway. Since IDO is expressed in various tissues, while TDO is localized almost exclusively in the liver, IDO appears to play a much more significant role in the kynurenine pathway than TDO [1-3]. Under physiologic conditions, IDO is expressed modestly, but it is highly induced by bacterial and viral infections [4].

Several lines of evidence from an experimental mouse model showed that IDO had immmunoregulatory potential. First, placental IDO plays a crucial role in maternal-tolerance of the fetus [5,6]. Second, the activity of IDO results in significant prolongation of graft survival of allografted pancreas islets [7,8]. Finally, T cell-mediated experimental asthma is inhibited by the up-regulation of pulmonary IDO [9]. With respect to tumors, one of the mechanisms concerning tumor immune escape might be IDO-dependent. Indeed, animal tumors expressing a higher level of IDO effectively escape from immune surveillance of the host by degrading local tryptophan, which in turn inhibits T cell responses [10,11]. Furthermore, IDO expression has been documented in multiple solid human tumors, such as brain, breast, lung, thyroid, liver, pancreas, colon, rectum, kidney, bladder, prostate, ovarian, cervix, endometrium, and skin tumors [10,12-21]. In addition, IDO is expressed in tumor-draining lymph nodes (TDLN). It is widely thought that either or both of these sites of IDO expression serve to inhibit immune responses to tumors, although the supporting evidence is insufficient [22-25].

Based on the immunoregulatory effect of IDO, the anti-IDO therapeutic approach has been under investigation. Preliminary results have revealed that inhibition of IDO could delay tumor progression in a combined setting [26-29]. However, one should bear in mind that IDO exhibits multiple activities. For example, the kynurenine pathway is essential for cell biosynthesis. The IFN-γ-induced IDO-dependent antimicrobial effect against *Toxoplasma gondii, Chlamydia psittaci*, and group B streptococcal infections is postulated to be secondary to the degradation of the essential amino acid, L-tryptophan [30]. Additionally, the role of IDO in the antitumor activity of human IFN-γ remains controversial [31-33]. Therefore, more attention should be paid to the effect of IDO inhibition [34-36]. To test whether IDO activity in cancer cells and TDLN participates synchronously in tumor progression, the current study analyzed the expression of IDO in cancer cells from primary tumor and lymph node metastases, in normal epithelium from adjacent non-cancerous tissues, and in immune cells from TDLN without tumor involvement in colon cancer. The results showed the paradoxical patterns of expression of IDO; specifically, both a higher density of IDO^+^cells in TDLN and down-regulation of IDO in metastatic cancer cells were associated with poor prognosis in colon cancers.

## Methods

### Materials

Seventy-one cases of pathologically-confirmed specimens were obtained from colon cancer patients who underwent radical resection between January 2000 and December 2000 in the Cancer Center of Sun Yat-Sen University, Guangzhou, China (Table 1). All of the patients were treated with 5-FU-based adjuvant chemotherapy postoperatively for 6 months. Patients were evaluated every 3 months during the 1^st ^year, every 6 months in the 2^nd ^year, and by telephone or mail communication once every year thereafter, for a total of 5 years. If recurrence or metastasis occurred, 5-FU-based chemotherapy was given according to the NCCN guideline. Overall survival was defined as the time from surgery to death; alternatively, censoring was done at the last known date the patient was alive.

**Table 1:** Patient Characteristics (N = 71)

Characteristic	No. of patients(%)
Age, years	
< 60	44(62.0)
≥ 60	27(38.0)
Gender	
Male	46(64.8)
Female	25(35.2)
T stage	
T1	1(1.4)
T2	15(21.1)
T3	54(76.1)
T4	1(1.4)
N stage	
N0	27(38.0)
N1–3	44(62.0)
M stage	
M0	58(81.7)
M1	13(18.3)

### Immunohistochemical assay and scoring systems

The expression of IDO in primary tumors matched with adjacent non-cancerous tissues, lymph node involvement, and TDLN without tumor metastases was determined with an immunohistochemical assay. The expression of Bin1 in primary tumors matched with adjacent non-cancerous tissues was also determined with an immunohistochemical assay. Briefly, formalin-fixed, paraffin-embedded archived tissues were cut into 4-μm sections. Then, the sections were dewaxed, rehydrated, blocked with hydrogen peroxide, and antigen was retrieved in a microwave in 10 mM citrate buffer (pH 6.0) for 10 minutes and cooled to room temperature. After blocking with 1% rabbit (or sheep) serum, the sections were incubated with sheep polyclonal antibody against human IDO at a dilution of 1: 50 (Hycult Biotechology, Uden, The Netherlands) or mouse monoclonal antibody against human Bin1 at a dilution of 1:100 (Millipore Corporation, MA, USA) overnight at 4°C, followed by biotinylated secondary antibody and streptavidin-biotinylated horseradish peroxidase complex. The sections were developed with diaminobenzidine tetrahydrochloride (DAB) and counterstained with hematoxylin. Negative controls were made with primary antibody replaced by PBS.

The following two scoring systems were used: 1) determination of the expression of IDO and Bin1 in cancer cells and normal epithelium; and 2) determination of the density of IDO^+ ^immune cells in TDLN. Each section was scored independently by two pathologists. If inconsistency existed, a third pathologists served to achieve consensus. In tumor and normal epithelium, the expression of IDO and Bin1 was interpreted for immunoreactivity using the 0–4 semi-quantitative system of Gastl [37] for both the intensity of staining and the percentage of positive cells (labeling frequency percentage). The intensity of membrane staining was grouped into the following 4 categories: no staining/background of negative controls (score = 0), weak staining detectable above background (score = 1), moderate staining (score = 2), and intense staining (score = 3). The labeling frequency was scored as 0 (≤ 5%), 1 (5% to 25%), 2 (26% to 50%), 3 (51% to 75%), and 4 (≥ 76%). The product index was obtained by multiplying the intensity and percentage scores, as follows: (-), (+), (++), and (+++) indicated sum indexes of 0~2, 3~5, 6~8, and 9~12, respectively; (-) and (+) were defined as no or modest expression, and (++) and (+++) were defined as strong expression.

For the density of IDO^+^cells in TDLN, another scoring system was used. Slides were examined under a low power (× 40~×100) microscope to identify the regions containing the highest percentage of IDO^+^cells (hot spot) in the lymph nodes. Five fields of hot spots within the lymph nodes were selected under a higher power (×200) microscope, and the average number IDO^+^cells in each field was calculated. The cases with ≤ 50 IDO^+^cells in each field were considered to have a low density of IDO^+^cells in the TDLN, whereas the cases with > 50 IDO^+^cells in each field were considered to have a high density of IDO^+ ^cells in the TDLN.

### Statistical analysis

The levels of IDO and Bin1 between primary tumor and adjacent epithelium or the levels of IDO between primary tumors and matched lymph node metastases were analyzed with a chi-square test or Fisher's exact test. The correlation between IDO expression and Bin1 expression, or the correlation between IDO expression or Bin1 expression and TNM stages was analyzed with Spearman rank correlation. The following factors were assessed with both univariate and multivariate analyses to determine the influence on overall survival: T stage, N stage, M stage, IDO expression in the primary tumor, Bin1 expression in the primary tumor, and the density of IDO^+^cells in TDLN without metastases. Kaplan-Meier curves were used to estimate the distributions of those variables to survival and compared with the log-rank test. The Cox regression model was used to correlate assigned variables with overall survival. All statistical analyses were carried out using SPSS 13.0 software (SPSS Inc., Chicago, IL, USA). Statistical significance was assumed for a two-tailed *P *< 0.05.

## Results

### The patterns of expression of IDO and Bin1

**Figure 1:**
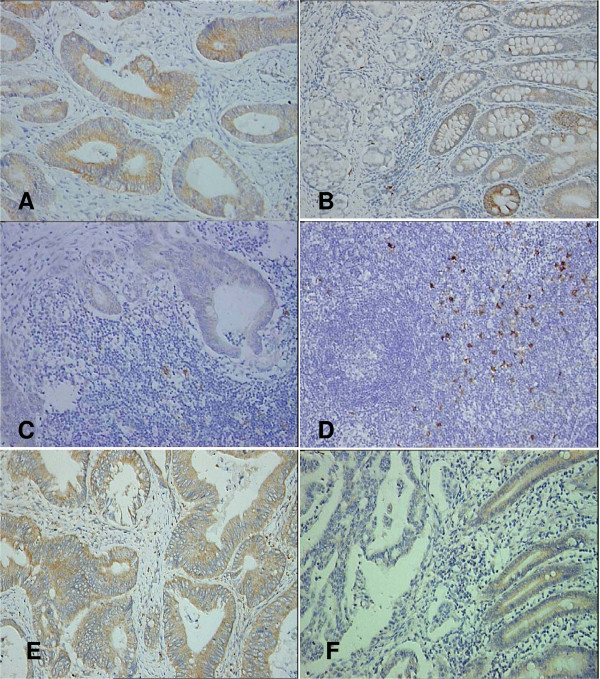
**Expression of IDO and Bin1 in colon cancer**. IDO is expressed in primary colon cancer cells (A), in adjacent non-cancerous epithelium (B) in lymph node metastases (C), and in tumor-draining lymph nodes without metastasis (D). Bin1 is also expressed in cancer cells and epithelium (E, F; immunohistochemical assay, ×200).

The expression of IDO was detected in the cytoplasm of tumor cells, normal epithelium, and immune cells. The staining of IDO occurred on both the luminal and basal surfaces of tumor cells and normal epithelial cells. In the same section, staining of IDO occurred both in cancer cells and normal epithelial cells (Fig. 1A, B). Similar staining of IDO also occurred in the tumor cells which had metastasized to the lymph nodes. As most of the sections lacked normal lymph tissue around the nests of cancer cells, the density of IDO^+^immune cells around the metastases was not available (Fig. 1C). The expression of IDO was detected on dendritic cell-like cells in the TDLN without metastases, which were derived from 27 patients without lymph node involvement (N0) and 33 patients with lymph node involvement (N1–3; Fig. 1D). According to the definition regarding the density of IDO^+^cells in TDLN (*vide supra*), 21 cases were grouped as high density IDO^+^cells in TDLN and 39 cases as low density of IDO^+^cells. Bin1 was also detected in the cytoplasm in the primary tumors and adjacent non-cancerous epithelium (Fig. 1E, F).

#### Comparison of the expression of IDO and Bin1 between primary tumors and lymph node metastases

Strong expression (++~+++) of IDO was documented in 9 of the 71 patients, while strong expression of Bin1 was documented in 33 cases. Since IDO stained both on the primary tumor cells and the normal epithelial cells, the levels of IDO between primary tumors and matched adjacent epithelium was compared; no difference existed (Table 2). The intensity of IDO between primary tumors and lymph node involvement was compared among the 41 cases which have matched primary tumors and lymph node involvements, and showed that the expression of IDO in lymph node involvement decreased (Table 3). The relationship between the levels of IDO and Bin1 was analyzed and failed to show any correlation (data not shown).

**Table 2:** The expression of IDO or Bin1 in primary tumors and matched adjacent non-cancerous epithelium (N = 71)

	IDO expression				*P *value	Bin1 expression				*P *value
	-	+	++	+++		-	+	++	+++	
Primary tumor (%)	40 (56.3)	22 (31.0)	6 (8.5)	3 (4.2)	0.936	21 (29.6)	17 (23.9)	19 (26.8)	14 (19.7)	0.350
Adjacent epithelium (%)	42 (59.2)	20 (28.1)	7 (9.9)	2 (2.8)		12 (16.9)	19 (26.8)	22 (31.0)	18 (25.3)	

**Table 3:** The expression of IDO in primary tumors and matched lymph node metastases (N = 41)

	IDO expression				*P *value
		
	-	+	++	+++	
Primary tumor (%)	18 (43.9)	15 (36.6)	6 (14.6)	2 (4.9)	0.020
Metastasis(%)	27 (65.9)	14 (34.1)	0	0	

#### Relationship between survival and TNM stages; the levels of IDO and Bin1 assessed with univariate survival analysis

**Figure 2:**
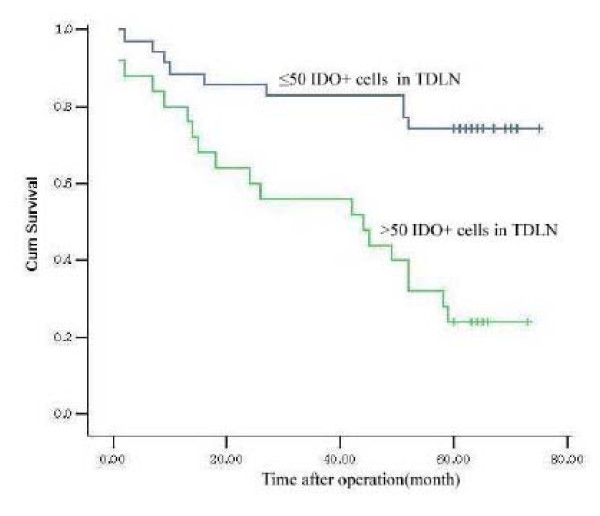
**The association of overall survival with the density of IDO^+^cells in TDLN in colon cancers**. The patients with a low density of IDO^+^cells in TDLN were strongly associated with a higher 5-year survival rate (73.1%) than the patients with a high density of IDO^+^cells (37.5%; Kaplan-Meier analysis, *P *< 0.05).

By the end of the 5-year follow-up, 39 patients were still alive, thus the 5-year survival rate in this group of patients was 55%. Before univariate and multivariate analyses, the correlation between the level of IDO or Bin1 with the TNM stages was analyzed. Neither IDO nor Bin1 expression correlated with TNM stages (data not shown). Then, the TNM stages, and the levels of IDO and Bin1 were analyzed with Kaplan-Meier survival analysis. The results showed that N stage, M stage, and the density of IDO^+^cells in TDLN indicated a poor prognosis, whereas the T stage and the levels of IDO and Bin1 in primary tumors were not related to survival. The patients with a higher density of IDO^+^cells in TDLN had a lower 5-year survival rate (37.5%) than the patients with a lower density of IDO^+^cells in TDLN (73.1%; Fig. 2).

#### Relationship between survival and the TNM stages, and the levels of IDO and Bin1 assessed with multivariate survival analysis

The Cox regression model revealed that patients with a higher N stage, a higher M stage, and a higher density of IDO^+^cells in TDLN without tumor involvement had a shorter survival, whereas no relationship was observed between the survival and T stage as well as the levels of IDO and Bin1 in primary tumors, indicating that in this group of patients the density of IDO^+^cells in TDLN without tumor involvement were independently prognostic (Table 4).

**Table 4:** The association between survival and TNM stages, and the levels of IDO and Bin1 in patients with colon cancers (N = 60)

Factors	B	SE	Wald	df	Sig.	Exp(B)	95%CI	
							
							Lower	upper
T stage	-.118	.389	.091	1	.763	.889	.415	1.906
N stage	.825	.258	10.220	1	.001	2.281	1.376	3.783
M stage	3.212	.579	30.821	1	.000	24.830	7.989	77.173
IDO expression in primary tumors	.598	.638	.878	1	.349	1.818	.521	6.345
Bin1 expression in primary tumors	-.241	.378	.405	1	.525	.786	.375	1.650
The density of IDO+cells in TDLN	1.321	.524	6.362	1	.012	3.746	1.342	10.452

## Discussion

To determine the role of IDO activity in the progression of colon cancers, this study analyzed the expression of IDO in tumor cells from primary tumors and lymph node metastases, in normal epithelial cells from non-cancerous tissues, and in immune cells from the TDLN. The results showed that a higher density of IDO^+^cells in TDLN was associated with a lower 5-year survival rate, which was a prognostic marker independent of TNM stage, although the T stage was not related to survival in this group of patients, which might derive from the obvious bias of T stage, with only one T1 and T4 patient studied. In contrast, as compared with non-cancer tissues, the increased expression of IDO was not observed in primary tumors. However, the decreased level of IDO was documented in lymph nodes metastases. These data suggest that the activity of IDO in tumor cells and immune cells differs.

Although the expression of IDO in TDLN has been observed in human tumors, the prognostic role of IDO in TDLN has only been documented in melanoma [24-27]. Among melanomas, the IDO^+^cells in TDLN appeared to be a population of plasmacytoid dendritic cell-like cells which blocked the initial response to tumor antigens, inhibited the ability of activated T cells to kill tumor cells, and enhanced the suppressive activity of T regulatory cells, resulting in local immunosuppression [38-42]. Except for melanoma, a highly immunogenic tumor, the prognostic effect of the density of IDO^+^cells in TDLN among other solid tumors, such as colorectal cancer, with modest immunogenicity is still elusive. This study revealed that the patients with a higher density of IDO^+^cells in TDLN had a lower 5-year survival rate (37.5%) than patients with a lower density of IDO^+^cells (73.1%), implying that the IDO in TDLN contributes to tumor progression, regardless of the immunogenicity of the primary tumors. Furthermore, this study also indicated that the IDO^+^cells in TDLN were not induced directly by the tumor cells as tumor cells were absent in these lymph nodes. The higher density of IDO^+^cells in TDLN might be ascribed to cancer cell-induced cytokines, exosomes, and tolerogenic dendritic cells which migrated from the primary tumor to the TDLN [43-48].

Considering the fact that IDO was also expressed in normal epithelium, the levels of IDO between primary tumors and its adjacent non-cancerous tissues were compared in this study. The results showed that no difference in IDO expression was observed. Secondly, as Bin1 could regulate the transcription of IDO, the Bin1 expression was also examined [49,50]. Again, no relationship between Bin1 expression and IDO expression was observed. Finally, neither IDO nor Bin1 in primary colon cancers was associated with the 5-year survival rate. Thus, these data suggested that it was less likely that the IDO activity in primary colon cancers contributed to tumor progression, which contrasted to the previous observation [13]. This discrepancy might derive from the research strategy as the IDO expression in the adjacent tissue might not been carefully evaluated in the previous study.

The expression of IDO in cancer cells is associated with a poor prognosis, as documented in multiple solid tumors. In this study, as the up-regulation of IDO was not observed in primary cancers, it is impossible to judge whether IDO activity is involved in tumor progression. Therefore, this study further compared the levels of IDO between primary tumors and matched lymph node metastases. Decreased expression of IDO was observed in lymph node metastases. Since lymph node involvement contributed to poor survival, decreased IDO in lymph node metastases do not support the hypothesis that IDO expressed by metastatic cancer cells contributes to metastasis by direct induction of local immunosuppression [51-55]. Therefore, these data suggest that IDO might have other potential than immunosuppression in metastatic colon cancer cells, although more evidence is needed to confirm this hypothesis. Based on the potential pluripotency of IDO, more specific IDO inhibitor might be needed for tumor therapy [56].

## Conclusion

This study observed the paradoxical patterns of IDO expression in colon cancer. One was the fact that a higher density of IDO^+^cells existed in TDLN, the other was the fact that the down-regulation of IDO occurred in metastatic colon cancer cells. This paradoxical phenomenon implied that IDO activity might contribute to the progression of colon cancer by multiple mechanisms including immunosuppression.

## Competing interests

The authors declare that they have no competing interests.

## Authors' contributions

WXJ, DY, ZX, PZZ, and WDS carried out the cases collection, GYF and PRQ carried out the immunohistochemical staining work and analysis, ZXS and LJ conceived the study, participated in its design and coordination, and helped draft the manuscript. All authors read and approved the final manuscript.

## References

[B1] Munn DH, Mellor AL (2007). Indoleamine 2,3-dioxygenase and tumor-induced tolerance. J Clin Invest.

[B2] Takikawa O (2005). Biochemical and medical aspects of the indoleamine 2,3-dioxy-genase-initiated L-tryptophan metabolism. Biochem Biophys Res Commun.

[B3] King NJ, Thomas SR (2007). Molecules in focus: indoleamine 2,3-dioxygenase. Int J Biochem Cell Biol.

[B4] Munn DH (2006). Indoleamine 2,3-dioxygenase, tumor-induced tolerance and counter-regulation. Curr Opin Immunol.

[B5] Munn DH, Zhou M, Attwood JT, Bondarev I, Conway SJ, Marshall B, Brown C, Mellor AL (1998). Prevention of allogeneic fetal rejection by tryptophancatabolism. Science.

[B6] Mellor AL, Munn DH (2004). IDO expression by dendritic cells: tolerance and tryptophan catabolism. Nat Rev Immunol.

[B7] Alexander AM, Crawford M, Bertera S, Rudert WA, Takikawa O, Robbins PD, Trucco M (2002). Indoleamine 2,3-dioxygenase expression in transplanted NOD Islets prolongs graft survival after adoptive transfer of diabetogenic splenocytes. Diabetes.

[B8] Jalili RB, Rayat GR, Rajotte RV, Ghahary A (2007). Suppression of islet allogeneic immune response by indoleamine 2,3 dioxygenase-expressing fibroblasts. J Cell Physiol.

[B9] Hayashi T, Beck L, Rossetto C, Gong X, Takikawa O, Takabayashi K, Broide DH, Carson DA, Raz E (2004). Inhibition of experimental asthma by indoleamine 2,3-dioxygenase. J Clin Invest.

[B10] Uyttenhove C, Pilotte L, Théate I, Stroobant V, Colau D, Parmentier N, Boon T, Eynde BJ Van den (2003). Evidence for a tumoral immune resistance mechanism based on tryptophan degradation by indoleamine 2,3-dioxygenase. Nat Med.

[B11] Zheng X, Koropatnick J, Li M, Zhang X, Ling F, Ren X, Hao X, Sun H, Vladau C, Franek JA, Feng B, Urquhart BL, Zhong R, Freeman DJ, Garcia B, Min WP (2006). Reinstalling antitumor immunity by inhibiting tumor-derived immuno-suppressive molecule IDO through RNA interference. J Immunol.

[B12] Ino K, Yoshida N, Kajiyama H, Shibata K, Yamamoto E, Kidokoro K, Takahashi N, Terauchi M, Nawa A, Nomura S, Nagasaka T, Takikawa O, Kikkawa F (2006). Indoleamine 2,3-dioxygenase is a novel prognostic indicator for endometrial cancer. Br J Cancer.

[B13] Brandacher G, Perathoner A, Ladurner R, Schneeberger S, Obrist P, Winkler C, Werner ER, Werner-Felmayer G, Weiss HG, Göbel G, Margreiter R, Königsrainer A, Fuchs D, Amberger A (2006). Prognostic value of indoleamine 2,3-dioxygenase expression in colorectal cancer: effect on tumor-infiltrating T cells. Clin Cancer Res.

[B14] Chen PW, Mellon JK, Mayhew E, Wang S, He YG, Hogan N, Niederkorn JY (2007). Uveal melanoma expression of indoleamine 2,3-deoxygenase: establishment of an immune privileged environment by tryptophan depletion. Exp Eye Res.

[B15] Sedlmayr P, Semlitsch M, Gebru G, Karpf E, Reich O, Tang T, Wintersteiger R, Takikawa O, Dohr G (2003). Expression of indoleamine 2,3-dioxygenase in carcinoma of human endometrium and uterine cervix. Adv Exp Med Biol.

[B16] Karanikas V, Zamanakou M, Kerenidi T, Dahabreh J, Hevas A, Nakou M, Gourgoulianis KI, Germenis AE (2007). Indoleamine 2,3-dioxygenase (IDO) expression in lung cancer. Cancer Biol Ther.

[B17] Pan K, Wang H, Chen MS, Zhang HK, Weng DS, Zhou J, Huang W, Li JJ, Song HF, Xia JC (2008). Expression and prognosis role of indoleamine 2,3-dioxygenase in hepatocellular carcinoma. J Cancer Res Clin Oncol.

[B18] Riesenberg R, Weiler C, Spring O, Eder M, Buchner A, Popp T, Castro M, Kammerer R, Takikawa O, Hatz RA, Stief CG, Hofstetter A, Zimmermann W (2007). Expression of indoleamine 2,3-dioxygenase in tumor endothelial cells correlates with long-term survival of patients with renal cell carcinoma. Clin Cancer Res.

[B19] Takao M, Okamoto A, Nikaido T, Urashima M, Takakura S, Saito M, Saito M, Okamoto S, Takikawa O, Sasaki H, Yasuda M, Ochiai K, Tanaka T (2007). Increased synthesis of indoleamine-2,3-dioxygenase protein is positively associated with impaired survival in patients with serous-type, but not with other types of, ovarian cancer. Oncol Rep.

[B20] Travers MT, Gow IF, Barber MC, Thomson J, Shennan DB (2004). Indoleamine 2,3-dioxygenase activity and L-tryptophan transport in human breast cancer cells. Biochim Biophys Acta.

[B21] Ino K, Yamamoto E, Shibata K, Kajiyama H, Yoshida N, Terauchi M, Nawa A, Nagasaka T, Takikawa O, Kikkawa F (2008). Inverse correlation between tumoral indoleamine 2,3-dioxygenase expression and tumor-infiltrating lymphocytes in endometrial cancer: its association with disease progression and survival. Clin Cancer Res.

[B22] Munn DH, Mellor AL (2006). The tumor-draining lymph node as an immune-privileged site. Immunol Rev.

[B23] Terness P, Chuang JJ, Opelz G (2006). The immunoregulatory role of IDO-producing human dendritic cells revisited. Trends Immunol.

[B24] Löb S, Königsrainer A (2008). Is IDO a key enzyme bridging the gap between tumor escape and tolerance induction?. Langenbecks Arch Surg.

[B25] Negin B, Panka D, Wang W, Siddiqui M, Tawa N, Mullen J, Tahan S, Mandato L, Polivy A, Mier J, Atkins M (2008). Effect of melanoma on immune function in the regional lymph node basin. Clin Cancer Res.

[B26] Yen MC, Lin CC, Chen YL, Huang SS, Yang HJ, Chang CP, Lei HY, Lai MD (2009). A novel cancer therapy by skin delivery of indoleamine 2,3-dioxygenase siRNA. Clin Cancer Res.

[B27] Ou X, Cai S, Liu P, Zeng J, He Y, Wu X, Du J (2008). Enhancement of dendritic cell-tumor fusion vaccine potency by indoleamine-pyrrole 2,3-dioxygenase inhibitor, 1-MT. J Cancer Res Clin Oncol.

[B28] Miyazaki T, Moritake K, Yamada K, Hara N, Osago H, Shibata T, Akiyama Y, Tsuchiya M (2009). Indoleamine 2,3-dioxygenase as a new target for malignant glioma therapy. J Neurosurg.

[B29] Hou DY, Muller AJ, Sharma MD, DuHadaway J, Banerjee T, Johnson M, Mellor AL, Prendergast GC, Munn DH (2007). Inhibition of indoleamine 2,3-dioxygenase in dendritic cells by stereoisomers of 1-methyl-tryptophan correlates with antitumor responses. Cancer Res.

[B30] MacKenzie CR, Heseler K, Müller A, Däubener W (2007). Role of indoleamine 2,3-dioxygenase in antimicrobial defence and immuno-regulation: tryptophan depletion versus production of toxic kynurenines. Curr Drug Metab.

[B31] Burke F, Knowles RG, East N, Balkwill FR (1995). The role of indoleamine 2,3-dioxygenase in the anti-tumour activity of human interferon-gamma in vivo. Int J Cancer.

[B32] Gasparri AM, Jachetti E, Colombo B, Sacchi A, Curnis F, Rizzardi GP, Traversari C, Bellone M, Corti A (2008). Critical role of indoleamine 2,3-dioxygenase in tumor resistance to repeated treatments with targeted IFNgamma. Mol Cancer Ther.

[B33] Melichar B, Hu W, Patenia R, Melicharová K, Gallardo ST, Freedman R (2003). rIFN-gamma-mediated growth suppression of platinum-sensitive and -resistant ovarian tumor cell lines not dependent upon arginase inhibition. J Transl Med.

[B34] Karanikas V, Speletas M, Zamanakou M, Kalala F, Loules G, Kerenidi T, Barda AK, Gourgoulianis KI, Germenis AE (2008). Foxp3 expression in human cancer cells. J Transl Med.

[B35] Slingluff CL, Speiser DE (2005). Progress and controversies in developing cancer vaccines. J Transl Med.

[B36] Nagorsen D, Voigt S, Berg E, Stein H, Thiel E, Loddenkemper C (2007). Tumor-infiltrating macrophages and dendritic cells in human colorectal cancer: relation to local regulatory T cells, systemic T-cell response against tumor-associated antigens and survival. J Transl Med.

[B37] Gastl G, Spizzo G, Obrist P, Dünser M, Mikuz G (2000). Ep-CAM overexpression in breast cancer as a predictor of survival. Lancet.

[B38] Sharma MD, Baban B, Chandler P, Hou DY, Singh N, Yagita H, Azuma M, Blazar BR, Mellor AL, Munn DH (2007). Plasmacytoid dendritic cells from mouse tumor-draining lymph nodes directly activate mature Tregs via indoleamine 2,3-dioxygenase. J Clin Invest.

[B39] von Bergwelt-Baildon MS, Popov A, Saric T, Chemnitz J, Classen S, Stoffel MS, Fiore F, Roth U, Beyer M, Debey S, Wickenhauser C, Hanisch FG, Schultze JL (2006). CD25 and indoleamine 2,3-dioxygenase are up-regulated by prostaglandin E2 and expressed by tumor-associated dendritic cells in vivo: additional mechanisms of T-cell inhibition. Blood.

[B40] Munn DH, Sharma MD, Hou D, Baban B, Lee JR, Antonia SJ, Messina JL, Chandler P, Koni PA, Mellor AL (2004). Expression of indoleamine 2,3-dioxygenase by plasmacytoid dendritic cells in tumor-draining lymph nodes. J Clin Invest.

[B41] Kahler DJ, Mellor AL (2009). T cell regulatory plasmacytoid dendritic cells expressing indoleamine 2,3 dioxygenase. Handb Exp Pharmacol.

[B42] Liu JY, Zhang XS, Ding Y, Peng RQ, Cheng X, Zhang NH, Xia JC, Zeng YX (2005). The changes of CD4+CD25+/CD4+ proportion in spleen of tumor-bearing BALB/c mice. J Transl Med.

[B43] Gajewski TF, Meng Y, Blank C, Brown I, Kacha A, Kline J, Harlin H (2006). Immune resistance orchestrated by the tumor microenvironment. Immunol Rev.

[B44] Zou W (2006). Regulatory T cells, tumour immunity and immunotherapy. Nat Rev Immunol.

[B45] Watanabe S, Deguchi K, Zheng R, Tamai H, Wang LX, Cohen PA, Shu S (2008). Tumor-induced CD11b+Gr-1+ myeloid cells suppress T cell sensitization in tumor-draining lymph nodes. J Immunol.

[B46] Polak ME, Borthwick NJ, Gabriel FG, Johnson P, Higgins B, Hurren J, McCormick D, Jager MJ, Cree IA (2007). Mechanisms of local immuno-suppression in cutaneous melanoma. Br J Cancer.

[B47] Ichim TE, Zhong Z, Kaushal S, Zheng X, Ren X, Hao X, Joyce JA, Hanley HH, Riordan NH, Koropatnick J, Bogin V, Minev BR, Min WP, Tullis RH (2008). Exosomes as a tumor immune escape mechanism: possible therapeutic implications. J Transl Med.

[B48] Huber V, Filipazzi P, Iero M, Fais S, Rivoltini L (2008). More insights into the immunosuppressive potential of tumor exosomes. J Transl Med.

[B49] Fallarino F, Gizzi S, Mosci P, Grohmann U, Puccetti P (2007). Tryptophan catabolism in IDO+ plasmacytoid dendritic cells. Curr Drug Metab.

[B50] Muller AJ, DuHadaway JB, Donover PS, Sutanto-Ward E, Prendergast GC (2005). Inhibition of indoleamine 2,3-dioxygenase, an immunoregulatory target of the cancer suppression gene Bin1, potentiates cancer chemotherapy. Nat Med.

[B51] Prendergast GC (2008). Immune escape as a fundamental trait of cancer: focus on IDO. Oncogene.

[B52] Witkiewicz A, Williams TK, Cozzitorto J, Durkan B, Showalter SL, Yeo CJ, Brody JR (2008). Expression of indoleamine 2,3-dioxygenase in metastatic pancreatic ductal adenocarcinoma recruits regulatory T cells to avoid immune detection. J Am Coll Surg.

[B53] Zamanakou M, Germenis AE, Karanikas V (2007). Tumor immune escape mediated by indoleamine 2,3-dioxygenase. Immunol Lett.

[B54] Yoshida N, Ino K, Ishida Y, Kajiyama H, Yamamoto E, Shibata K, Terauchi M, Nawa A, Akimoto H, Takikawa O, Isobe K, Kikkawa F (2008). Overexpression of indoleamine 2,3-dioxygenase in human endometrial carcinoma cells induces rapid tumor growth in a mouse xenograft model. Clin Cancer Res.

[B55] Battaglia A, Buzzonetti A, Baranello C, Ferrandina G, Martinelli E, Fanfani F, Scambia G, Fattorossi A (2009). Metastatic tumour cells favour the generation of a tolerogenic milieu in tumour draining lymph node in patients with early cervical cancer. Cancer Immunol Immunother.

[B56] Löb S, Königsrainer A, Rammensee HG, Opelz G, Terness P (2009). Inhibitors of indoleamine-2,3-dioxygenase for cancer therapy: can we see the wood for the trees?. Nat Rev Cancer.

